# The Role of BCL-2 and PD-1/PD-L1 Pathway in Pathogenesis of Myelodysplastic Syndromes

**DOI:** 10.3390/ijms24054708

**Published:** 2023-03-01

**Authors:** Bartłomiej Kuszczak, Tomasz Wróbel, Katarzyna Wicherska-Pawłowska, Justyna Rybka

**Affiliations:** Department and Clinic of Hematology, Blood Neoplasms and Bone Marrow Transplantation, Wroclaw Medical University, Ludwika Pasteura 4 Street, 50-367 Wroclaw, Poland

**Keywords:** myelodysplastic syndromes, BCL-2, MCL-1, PD-1, PD-L1, BH3-mimetics

## Abstract

Myelodysplastic syndromes (MDSs) belong to a group of clonal bone marrow malignancies. In light of the emergence of new molecules, a significant contribution to the understanding of the pathogenesis of the disease is the study of the B-cell CLL/lymphoma 2 (BCL-2) and the programmed cell death receptor 1 (PD-1) protein and its ligands. BCL-2-family proteins are involved in the regulation of the intrinsic apoptosis pathway. Disruptions in their interactions promote the progression and resistance of MDSs. They have become an important target for specific drugs. Bone marrow cytoarchitecture may prove to be a predictor of response to its use. The challenge is the observed resistance to venetoclax, for which the MCL-1 protein may be largely responsible. Molecules with the potential to break the associated resistance include S63845, S64315, chidamide and arsenic trioxide (ATO). Despite promising in vitro studies, the role of PD-1/PD-L1 pathway inhibitors has not yet been established. Knockdown of the PD-L1 gene in preclinical studies was associated with increased levels of BCL-2 and MCL-1 in lymphocytes T, which could increase their survival and promote tumor apoptosis. A trial (NCT03969446) is currently underway to combine inhibitors from both groups.

## 1. Introduction

Myelodysplastic syndromes (MDSs) belong to a group of clonal bone marrow malignancies. According to the 2016 WHO classification, the criteria for the diagnosis of the disease include peripheral cytopenias in at least one lineage, features of dysplasia in ≥10% of cells, characteristic cytogenetic changes and a blast percentage of 5–19% [1]. The incidence of MDS ranges from 3.2 to 12.4/100,000/year. An estimated 86.4% of diagnoses are in patients >60 years of age, and the median age at diagnosis is 76 years [2,3,4]. In addition to age, risk factors for MDSs include alkylating molecules, antimetabolites, certain immunosuppressive drugs (such as azathioprine) and exposure to ionizing radiation [5]. The effectiveness of the most commonly used hypomethylating agents (HMA) is limited. Only 40–50% of patients respond to treatment, and complete remission (CR) is achieved by 10–20%. When the response to HMA is lost, the median survival is 4.5 months [6]. The advanced age of patients and age-related comorbidities mean that <10% of patients are eligible for allogeneic hematopoietic stem cell transplantation (alloHSCT) [7,8]. 

The pathogenesis of MDSs is complex. One of the factors involved is genetic instability. Approximately 78% of MDS patients are thought to have at least one somatic mutation [9]. These include both cytogenetic aberrations (the most common are −5/5q, −7/7q-, +8, 20q-, +21, 12p-, 13q- and 17p-), detected in 40–60% of cases, as well as molecular (UTX, SF3B1, U2AF1, TP53 and RUNX1) and epigenetic (DNMT3A, TET2, IDH1/2 and ASXL1) changes. Although many of these have prognostic significance, and although the last group has become relevant to the application of HMA [6], they do not fully explain the complexity of the tumor, nor do they take into account its interaction with the environment [10,11]. Non-genetic aspects also play an important role in the formation and course of MDSs. In light of the emergence of new molecules successfully used in the treatment of bone marrow malignancies [12,13,14], the study of BCL-2, PD-1, programmed cell death receptor 1 ligand (PD-L1) and programmed cell death receptor 2 (PD-L2) can make a significant contribution to the understanding of the disease.

## 2. Function of the BCL-2 Family of Proteins

BCL-2 is the first anti-apoptotic protein of the family of the same name, which is significantly involved in the regulation of cell apoptosis. It is mutated through translocation (14;18) in follicular lymphoma (FL) cells [15]. In the BCL-2 family, we distinguish anti-apoptotic proteins (BCL-2, MCL1, BCL-XL, BFL1/A1, BCL-W and BCL-2L10) composed of three BCL-2 homologous domains (BH) and a transmembrane domain (TM). They are found on the outer mitochondrial membrane or in the cytosol and may sometimes be found on other organelles such as the endoplasmic reticulum. Proapoptotic proteins, on the other hand, are divided into those with a single BH3 domain (BID, BIM, PUMA, BAD, BIK, HRK, NOXA and BMF) and those with multiple domains (BAX, BOK and BAK). BAX and BAK (known as effectors), when activated by proteins with a single domain (BH3-only) oligomerize to form channels in the mitochondrial membrane [16]. This leads to the permeabilization of the outer mitochondrial membrane and the release of apoptogenic factors, including cytochrome c, procaspases, Omi/HtrA2 protease, Smac/DIABLO protein, AIF or endonuclease G. Ultimately, this causes cell death through the intrinsic apoptotic pathway [17,18]. The role of the BCL-2 protein in this process is to bind to BAX and BAK and thus inhibit the process. Additional regulation of the whole system is provided by some BH3-only proteins, called sensitizer proteins—they do not directly affect BAX and BAK effectors but operate through the inhibition of BCL-2 [17]. Physiologically, the BCL-2 protein is essential for lymphocyte maturation. It is highly expressed during the differentiation of T cells into cells with single antigen expression (CD4−, CD8+ or CD4+ and CD8−) and memory T cells [19].

Initially, it was suggested that BCL-2 promotes the growth and proliferation of cancer cells; however, more detailed studies on transgenic mice overexpressing the BCL-2 protein showed that the protein only inhibits apoptosis and has no effect on cell proliferation. Despite the fact that it is not involved in all processes leading to cell death (in most cases, it did not inhibit the action of the FAS death receptor), it is able to desensitize it to anticancer drugs [20]. 

## 3. Genetic Background, Structure and Polymorphism

The BCL-2 gene is located on the long arm of chromosome 18 (18q21.33). It consists of 720 base pairs, forming three exons and two promoter regions (P1 and P2) [21]. P1 is responsible for initiating over 95% of transcription [22]. It potentially encodes two proteins, BCL-2α and BCL-2β, which differ in the presence of a transmembrane domain in the α subtype. The protein itself consists of eight α-helixes, two of which (α5 and α6) form a hydrophobic nucleus and are surrounded by the others. In another view, the protein is divided into four BH domains, the first three of which (BH1, BH2 and BH3) form a pocket that connects through the BH3 domain of other proteins in the family and interacts with them. The BH4 domain is suspected to have anti-apoptotic activity [23]. 

Polymorphisms of the BCL2 gene described in the literature include rs2279115, rs1801018 and rs1564483 variants. Two meta-analyses conducted have shown that the presence of the rs2279115 variant in the promoter region is associated with a higher risk of cancer among Asians but not Caucasians. However, data from the meta-analyses did not agree on the prognostic value of this option, nor did they include MDSs. There are no publications in the available literature describing the significance of BCL-2 polymorphisms in MDSs [22,24,25]. Therefore, knowledge of the variability of BCL-2 polymorphisms may be important to better understand the clinical course of MDSs.

## 4. The Importance of the BCL-2 Family in the Course of MDS

Two antagonistic processes are involved in the pathogenesis of MDSs. On one hand, there is the excessive, uncontrolled proliferation of cancerous blasts. On the other hand, there is increased apoptosis. Disruption of this peculiar “pathological balance” is thought to be the cause of progression to acute myeloid leukemia, or the onset of MDS with bone marrow depletion (a type specific to the pediatric population) [26,27]. The number of CD34+ cells undergoing apoptosis is highest in low-risk MDSs (Lr-MDSs) and lowest after transformation into acute myeloid leukemia (AML) [28]. The possibility of learning about new potential markers that could be predictors of disease progression seems important. Contrary to predictions based on IPSS, some patients with an Lr-MDS achieved shorter progression-free survival and overall survival (OS). Increased BCL-2 activity is considered to be one potential factor in this clinical situation [29,30]. The presence of BCL-2 family proteins, although significantly more strongly expressed in advanced MDSs, did not become an independent prognostic factor because of the correlation that occurred with the number of blasts and the type of MDS according to FAB. However, it was noted that the ratio of pro-apoptotic proteins (BAX/BAD) to anti-apoptotic proteins (BCL-2/BCL-XL) in CD34+ cells is higher in Lr-MDSs than in healthy controls and decreases as they evolve to higher stages. Increased BCL-2 expression is correlated with chromosome 7 aberrations that could inhibit blast apoptosis. A possible explanation for this process is the potential activation of proapoptotic mechanisms at low stages and the loss of their function as the MDS progresses [28]. This suggests that impaired expression of BCL-2 family proteins significantly affects disease progression and resistance. 

This seems to be confirmed by the observations of Vidal et al. They conducted a retrospective study on a group of 70 patients diagnosed with MDS, determining the importance of BCL-2-like protein 10 (BCL-2L10). In patients with an HMA resistance, 11% (median) of bone marrow cells showed BCL-2L10 expression compared to 1% (median) in the HMA-sensitive group. A significant increase in BCL-2L10 expression was noted during treatment and was higher in the group refractory to treatment. A higher percentage of cells showed an inverse correlation with patients achieving CR, taking 10% BCL-2L10 positive cells as the cutoff point. The median survival for patients was 9 months (>10% BCL-2L10) and 15.6 months (<10% BCL-2L10).

An important and, in recent years, increasingly well-understood member of this family of proteins is the myeloid leukemia-1 (MCL-1) protein that is mentioned in the introduction. Overexpression of MCL-1 has been observed in numerous myeloid and solid tumors [31,32]. It involves an imbalance between proapoptotic and anti-apoptotic mechanisms. Its function is based on its potential to hetero-oligomerize with pro-apoptotic BCL-2 family members (BIM, BAK, NOXA, PUMA or BID) and neutralize them. In vivo studies on a resistant blast cell line (OCI-AML3) showed its increased levels and prolonged stability after blocking the BCL-2 protein. This resulted in the prolonged survival of cancer cells. Due to its different structure compared to BCL-2 and BCL-XL (the absence of the BH4 domain and the presence of unusual amino acid residues in the BH3 domain) but similar function, it may represent a clinically relevant alternative pathway for anti-apoptotic signaling, causing resistance. This is supported by the observation that when the MCL-1 gene was turned off (knockdown), the survival time of the tested cells was shortened [18,33,34]. MCL-1 could become an important targeting point for targeted molecules. However, the clinical use of this mechanism could be dangerous and problematic as this protein is crucial for the survival of vital cells, such as cardiomyocytes and neurons [20]. 

The significance of the proapoptotic BCL-2 ovarian killer (BOK) protein in MDSs is complex and not fully elucidated. Its role is to put the cell into the apoptosis pathway as a result of exposure of the endoplasmic reticulum to stress factors [35]. On the one hand, there are reports of BOK’s role as an inhibitor of solid tumor growth, while on the other hand, its leukemogenic potential has been suggested [36]. Seong-Ho Kang et al. conducted a study on Nup98-HoxD13 (NHD13) transgenic mice, which developed MDS/AML due to the t(2;11)(q31;p15) translocation. After knockdown of the bok gene in transgenic mice, the authors expected a delay in the progression of the MDS to AML based on previous experience. However, in the course of their study, they found no differences between NHD13 and NHD13/bok−/− mice. However, they noted that the NHD13/bok−/− mice developed progressive anemia relative to the control group, and that their erythrocytes were hypochromic and had macrocytic morphology. The granulocytic and thrombocytic lines did not differ from the controls [37]. Thus, the role of the BOK in the MDS is complex and requires further research. Based on the above observations, it appears to be primarily responsible for maintaining relatively normal erythropoiesis in MDSs.

Literature reports on the role of the BAD protein in MDSs are scarce. The results obtained by Yasuko Hamada et al. appear to be clinically relevant; they found increased CD7+ expression correlating with decreased BAD levels in blasts. They showed that the combination of IPSS-R and increased CD7+ expression is a strong predictive factor associated with shortened patient survival [38].

## 5. New Targeted Molecules, Therapeutic Perspectives and Clinical Use

Over the years, an increasing understanding of the BCL-2 family has begun to direct research into attempting to translate the increasingly extensive theoretical knowledge into practical, clinical use. The importance of BCL-2 in oncogenesis has been recognized. The search began for targeted molecules that could intervene directly in the entire system, restoring its balance and thereby inhibiting tumor growth.

The first attempts made were to create antisense nucleotides directed against mRNAs containing information about individual family proteins. In vitro and preclinical in vivo studies tested antisense nucleotides against BCL-XL and BCL-2/BCL-XL. The first molecule to advance to the clinical trial phase was oblimersen (genasense, G3139) [31]. Oblimersen binds to the first six codons of the mRNA that carries the coding sequence for BCL-2. It then activates ribonuclease H, which hydrolyzes the mRNA strand, thus blocking the formation of the protein before the translation step [39]. Reports on the use of oblimersen for the treatment of MDS are scarce. However, in 2021, results from a phase III trial for the treatment of AML in a population ≥ 60 years old were published. The 506 patients were randomized into two groups: one received standard treatment based on daunorubicin and cytarabine with the addition of oblimersen, and the other received only the standard treatment. Considering all participants, there were no significant differences in CR, OS, DFS or EFS rates between those who received G3139 and those who did not, regardless of genetic risk group according to ELN. However, special attention should be paid to the group of patients with AML preceded by MDS (97 patients). In this population, a prolonged disease-free period with little toxicity was shown in the oblimersen group [40]. The study was discontinued due to the lack of evidence of the molecule’s clinical efficacy and the difficulties associated with the need to administer the drug by continuous infusion [19]. The hypothesis that due to its different point of entry from other BCL-2 inhibitors, oblimersen could potentially exhibit synergistic effects with them in MDSs, has not yet been tested. This may warrant and encourage additional studies on the efficacy of this molecule in MDS patients. 

There are few scientific reports on the effect of ATO (especially in combination with decitabine) on the induction of apoptosis in MDS in vitro [41,42,43]. In a study by Galimberti et al., in which a combination of ATO and ascorbic acid was used, an increased expression of pro-apoptotic BAD and BAX and decreased expression of anti-apoptotic BCL2L10 were noted in patients who responded to the treatment [44]. A limitation of the study was the small study group (12 people). ATO increases the stress exerted on the endoplasmic reticulum, which is one of the factors that induces cell apoptosis involving BCL-2 proteins [45,46]. However, large-group studies confirming the efficacy of ATO in MDSs have not been conducted. Currently, it seems that the interest of the scientific community is directed towards another promising group of drugs: BH3 mimetics. 

Obatoclax (GX15-070) belongs to the BH3 mimetics group and is a Pan-BCL-2 inhibitor with affinity for BCL-2, BCL-XL, BCL-w and MCL-1. Its proapoptotic effects include the separation of BIM and BAK from BCL-2 and MCL-1 and the formation of an active BAK/BAX complex. In vitro, it showed promise in breaking the resistance associated with MCL-1 overexpression [47]. In a Phase I study of 14 patients diagnosed with MDS, three patients achieved improvement of transfusion dependence with good treatment tolerance. The most commonly reported adverse effects (AEs) were mild neurological disturbances including drowsiness, dizziness, and euphoria. A reduction in transfusion dependence was also achieved. Unfortunately, in a multicenter Phase II study on 24 patients with untreated MDS, the results were not satisfactory, and it was decided to discontinue the study. The response rate was 8%, with thrombocytopenia, anemia and pneumonia among the Grade 3–4 AEs. In addition, the molecule was administered in continuous infusions over 24 h, which placed a heavy burden on both the patient and staff [19].

ABT-737 and navitoclax (ABT-263) belong to the same group as the aforementioned obatoclax. ABT-737 showed an affinity two to three orders of magnitude higher for BCL-2, BCL-XL and BCL-W than the previously developed BH3 mimetics, raising hopes for it. In vitro studies on AML and MDS cell lines (HL 60, U 937, P39) demonstrated its ability to increase cytochrome C release and activate caspases, resulting in cell apoptosis in two of the three cell lines [48]. The results of in vivo experiments many years later prolonged the OS of high-risk MDS (HR-MDS) mice by initiating apoptosis in cells that initiate progression to AML [49]. Due to the unfavorable solubility of ABT-737, the molecule has not been used in clinical practice. This problem was decided to be solved by modifying ABT-737, increasing its bioavailability and thus making it possible to take the molecule orally. Thus, studies were initiated on navitoclax; however, the navitoclax caused dose-dependent thrombocytopenia through BCL-XL inhibition. This has limited its clinical application [50,51].

Of all the BH3 mimetics, venetoclax is the most interesting and promising in the scientific community. Unlike other molecules in this group, it is a highly selective BCL-2 inhibitor. Lacking an affinity for BCL-XL, it has less tendency to induce apoptosis of thrombopoietic lineage cells and cause life-threatening bleeding [19]. ASXL1, RUNX1, EZH2 and TP53 mutations, which worsen prognosis in MDSs, did not affect BCL-2 expression. Therefore, BCL-2 inhibition could effectively induce blast apoptosis in HR-MDSs. In vitro results showed the efficacy of venetoclax in cells with an unfavorable genetic profile, which led to interest in its potential to treat relapsed and refractory disease [52]. In vitro studies have shown a synergistic effect of HMAs being the standard of care for HR-MDS with venetoclax, even for MDSs refractory to HMA monotherapy [52,53]. For this reason, most of the clinical trials that have been conducted have been based on the combination of azacitidine (AZA) and venetoclax. In a Phase Ib study (NCT02942290) on a population of 59 patients with HR-MDS, doses of venetoclax were escalated in combination with AZA. A dose of 400 mg was established as a safe dose that demonstrates efficacy. Treatment toxicity was acceptable, and the most common adverse reactions included gastrointestinal disorders and cytopenias. Among serious adverse effects (SAEs), neutropenic fever was the most common (31% of patients). Of the patients evaluated, 50% achieved improvement in morphology parameters (another study confirms that the combination of venetoclax with AZA is selectively toxic to blasts in relapsed and refractory MDS (Rr-MDS) but preserves normal hematopoiesis) [54]. CR was achieved in 18 patients, mCR in 22, disease stabilization (DS) in 11 and progression in 2 [55]. In a study by Garcia et al., AZA was also combined with venetoclax. A population of 78 patients with HR-MDS initially received venetoclax in doses of 400 mg and 800 mg; however, due to high toxicity it was decided to titrate the drug (100 mg, 200 mg and 400 mg). The overall response rate (ORR) was 77%, including CR 42% and mCR 35% [54].

The next step was the initiation of the randomized, double-blind, Phase III VERONA study of venetoclax vs. a placebo. It appears that the results of the above study may be crucial for the recognition of venetoclax as a standard of treatment for HR-MDS [56]. Clinical trials (NCT02966782 and NCT03404193) are also being conducted on the Rr-MDS group and are expected to be completed in 2022 and 2024, respectively. Preliminary results seem promising. ORR was achieved by 40% of subjects, the median time to response was 1.2 months, and the OS, assessed at 12 months, was 65% [56]. The molecule also appears to be applicable to the population eligible for alloHSCT. Adding it to the conditioning based on fludarabine and busulfan proved safe, and the molecule itself in the above combination proved active [57].

However, the expected response to venetoclax is not the same in all patient groups. Factors that can predict response to venetoclax are being sought in order to select patients the most promising response to the therapy used. With this approach, the idea of targeted treatment in oncology is fully realized. Increased levels of BCL-2 in cells assessed by flow cytometry may be a predictor of good response to venetoclax [58]. The architecture of bone marrow cells in MDSs seems to be important. An article by Ganan-Gomez et al. was published in Springer, in which bone marrow biopsy specimens from healthy donors were compared to those from MDS patients. On this basis, two populations were identified in the second group: one (52%) with a disturbed differentiation pattern expressed by an increased percentage of common myeloid progenitor cells (CMP) and another (48%) with an increased percentage of granulocytic-monocytic progenitor cells (GMP). A differential gene expression analysis of CMP and GMP isolated from MDS samples revealed a significantly higher expression of genes whose constitutive activation is associated with impaired marrow differentiation and apoptosis. A CMP-predominant MDS showed a better response to venetoclax than a GMP-predominant MDS despite the fact that BCL-2 expression was similar in both groups. This suggests that the GMP-mediated apoptosis of MDS cells relies on different pathways than the one regulated by BCL-2 [11]. This hypothesis requires further research. However, if confirmed, the assessment of bone marrow cytoarchitecture could become an important decision-making tool in choosing the optimal therapy. 

Venetoclax may find its application in the group of patients receiving therapy related MDS (t-MDS), which is characterized by a particularly unfavorable prognosis. In a retrospective study conducted by Shah et al. on 378 patients with diagnosis of therapy-related myeloid neoplasms, 48 patients suffering from t-MDS received venetoclax. The first-line use of venetoclax has been shown to result in a longer OS (9.4 vs. 6.1 months). However, it did not affect PFS (6.1 vs. 6.1 months) when compared to HMA. The prognosis was worsened by chromosome 7 abnormalities. Neither the TP53 mutation nor the number of blasts at diagnosis influenced the prognosis. Importantly, no clonal selection with the TP53 mutation was observed after the use of venetoclax [59].

As previously mentioned, increased levels of the MCL-1 protein are a clinically relevant mechanism for achieving resistance of MDS cells to BCL-2 inhibitors. Expression of the MCL-1 gene increases significantly after treatment with BH3 mimetics, causing the cancer cell to form a specific bypass that omits the blocked proteins [18,33]. One way to break this kind of resistance may be to combine venetoclax with daunorubicin and cytosine arabinoside [34]. The targeted MCL-1 inhibitor molecule S63845 is proving to be effective and safe in many types of cancer. A Phase I study on the use of another MCL-1 inhibitor, S64315, in MDS and AML is also underway [60].The findings may pave the way for further research into further lines of treatment and therapy for BH3 mimetic-resistant disease.

**Figure 1:**
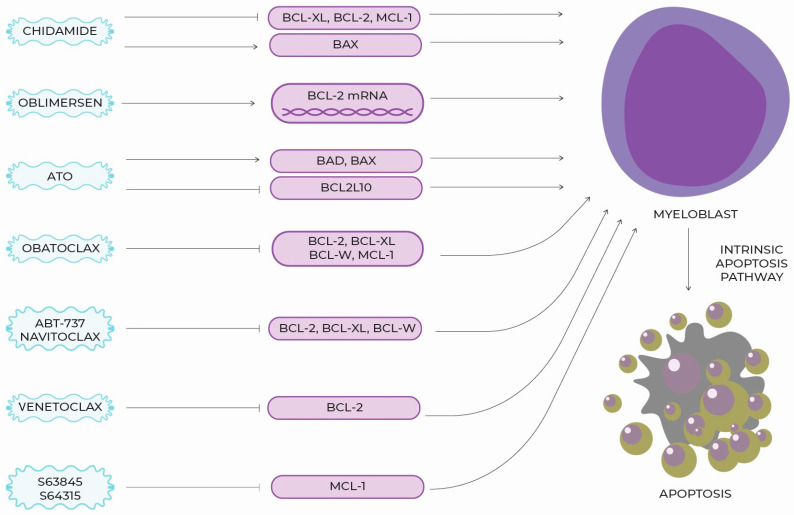
Recently tested particles with potential to interact with BCL-2 family and supporting cell apoptosis.

Chidamide is an oral benzamide group histone deacetylase (HDAC) inhibitor selectively active against HDAC1, HDAC2, HDAC3 and HDAC10 [61]. Previous studies by Chinese authors indicated that it induces apoptosis and arrests the cell cycle of MDS blasts at the G0/G1 stage. Its mechanism of action is complex, but blocking the JAK2/STAT3 signaling pathway appears to be responsible for its anti-tumor effects. Secondarily, the expression of BCL-xl, BCL-2 and MCL-1 decreases, while the expression of BAX increases [62,63]. Synergistic effects of chidamide with decitabine are observed. These molecules block the cell cycle in two different phases (G0/G1 and G2/M, respectively) [64]. In particular potential, is observed in lowering MCL-1 expression. This hypothesis is supported by the results of a study in which the combination of venetoclax and chidamide showed a synergistic effect. In vitro, it promoted blast apoptosis. In vivo, longer progression-free and survival times were achieved in mice receiving the combination treatment compared to those receiving monotherapy [65]. Research into the role of chidamide as a potential resistance-breaking molecule in MDS remains a scope for further research [Figure 1, Table 1].

## 6. BCL-2 Section Summary

The intrinsic apoptosis pathway is central to the pathogenesis of MDS. A more thorough understanding of the BCL-2 family of proteins crucial to its regulation has already yielded tangible benefits in the treatment of many cancers. However, it seems that the potential of this group of proteins has not yet been fully realized. Further studies are needed to determine the predictive value of BCL-2 gene expression as a factor that may indicate an increased risk of transformation to AML. It is worth highlighting the great potential of BH3 mimetics. It seems that current research should be directed at finding mechanisms and molecules to overcome resistance to the use of this group of drugs and finding a diagnostic standard to select patients who have the best chance of a good response to their use. 

## 7. PD-1—Structure and Function

The PD-1/PD-L1 signaling pathway is one of the most important components of the system that regulates the body’s normal immune response. Physiologically, it plays an inhibitory role in the cellular response, and its activation is associated with the normal selection and maturation of lymphocytes in the thymus. The PD-1 receptor (CD279), due to its structural similarity, has been assigned to the CD28 family and the Ig-like superfamily. Its presence has been described on activated T cells, B cells, NK cells, maturing thymocytes and some cells of the myeloid lineage. The gene encoding the PD-1 protein (pdcd-1) is located at locus 2q37.3 and consists of 5 exons. (62) Its product is a transmembrane protein consisting of 288 amino acids whose two hydrophobic domains, encoded by the third exon, are located in the cell membrane. Crucial to the function of the entire protein is a tyrosine located intracellularly in the proximal amino acid motif (ITIM immunoreceptor tyrosine-based inhibitory motif) [66]. In T cells, the activated PD-1 receptor affects multiple intracellular signaling pathways. Through tyrosine phosphorylation of ITIM, SHP-1 and SHP-2 are recruited: these block signaling pathways associated with T-cell receptor (TCR) and B-cell receptor (BCR), respectively. The signaling pathways mentioned include PKCδ, RAS-ERK1/2 and PI3K/AKT. This leads to reduced cytokine production, reduced proliferation and the promotion of apoptosis [67]. As a result, the energy metabolism of the cell is disrupted by increasing the activity of glycolytic enzymes. PD-1 is considered a potent inhibitor of T-cell activation. It is estimated that after its activation, the number of transcripts in a T lymphocyte decreases by 90%. Another mechanism of action of PD-1, which is no less important, is the inhibition of TCR signaling via the ZAP-70 kinase [68]. The role of PD-1 is to counteract the excessive response of the immune system. T lymphocytes with a strong stimulation of PD-1 receptors enter a state of exhaustion, leading to a progressive loss of their function and an inability to mount an immune response [69]. This phenomenon is well-documented in the course of chronic viral infections, such as hepatitis B and hepatitis C, and in the setting of cancerous tumors [70,71]. Mice with deletion of the pdcd-1 gene had autoimmune diseases such as lupus arthritis, glomerulonephritis, dilated cardiomyopathy and Type I diabetes [72,73,74]. Factors that regulate pdcd-1 gene expression include glucocorticosteroids, interleukins IL-2 IL-7 IL-12, IL-15, IL-18 and IL-21, IFN-γ and nuclear factor of activated T cells c1 (NF-ATc1) [75,76,77]. 

The most studied and best-known PD-1 polymorphisms include PD-1.1 (rs36084323), PD-1.3 (rs11568821), PD-1.5 (rs2227981), PD-1.9 (rs2227982) and PD-1 rs7421861. Based on a 2019 meta-analysis by Hashemi et al., the presence of the rs2227981 and rs11568821 polymorphisms may reduce the risk of certain solid tumors, while rs7421861 significantly increases the risk [78,79]. The significance of the above variants in MDS has not yet been understood. However, based on the results of the present study, they should draw the special attention of future researchers. 

## 8. PD-L1 and PD-L-2 Structure and Function

The PD-1 receptor ligands are PD-L1 (CD274) and PD-L2 (CD273). All the proteins mentioned belong to the same family of Ig-like transmembrane receptors. The former is present on both non-hematopoietic cells (endothelial, neurocytes, astrocytes, pancreatic islet cells and trophoblast cells) and hematopoietic cells as well as in inflamed tissues. In humans, it is induced by IFN-γ. Expression of the second is limited to antigen-presenting cells (macrophages and dendritic cells), mast cells and subtype B1 lymphocytes [73,80,81,82]. The formation of PD-1/PD-L1 and PD-1/PD-2L complexes is possible by linking analogous, IgV-like domains [83]. Among the known polymorphisms, the PD-1L rs4143815 variant has been linked to a higher risk of cancer [79].

## 9. The Role of the Pro-Inflammatory Microenvironment in the Pathogenesis of MDSs

The appearance of dysplastic cancerous blasts in the bone marrow is strongly associated with changes in the environment of the bone marrow niche and far-reaching immunological disorders, involving both the tumor microenvironment and, more broadly, the entire body [84,85]. There are two opposing but not mutually exclusive concepts describing the sequence of events in the development of MDSs. The first suggests that the acquisition of neoplastic features by blasts is initiated by the disruption of the marrow stroma. The described cytogenetic aberrations in the stroma cells differ from those found in neoplastic hematopoietic cells—this suggests that their origin is of a different origin than MDS cells. Deletion of the Dicer1 gene in osteoprogenitor cells was shown to lead to the development of MDS [86]. Thus, it is possible that mutations in the stroma cells cause the loss of its anti-cancer functions and support normal hematopoiesis, initiating the development of MDS. The second hypothesis suggests that genetic and metabolic abnormalities accumulate in cells exposed to direct contact with MDS cells, ultimately leading to dysfunction. Both hematopoietic and stromal cells are responsible for the increased secretion of vascular endothelial growth factor (VGEF), which is necessary for tumor growth [87].

The microenvironment of MDS secretes numerous cytokines, many of which have pro-inflammatory and immunomodulatory effects [88]. Chronic inflammation can result in the proliferation and activation of myeloid-derived suppressor cells (MDSCs) which secrete the pro-inflammatory protein S100A9. In a pathological feedback mechanism, this protein secondarily induces the expansion of MDSCs, leading to the apoptosis of hematopoietic and progenitor cells [89,90]. Increased levels of the S100A9 protein directly and indirectly (via the MYC protein) stimulate the expression of PD-1 and PD-L1 in the myeloid niche. This process is thought to be one of the elements responsible for the disappearance of normal hematopoiesis and the creation of an immunosuppressive environment, facilitating the escape of the tumor from immune surveillance [91].The interaction between PD-l and its ligand suppress cell proliferation and cytokine secretion dependent on TCR. In MDS patients, the CD4/CD8 ratio is disturbed due to the decreased number of CD4+ lymphocytes. [92] With prolonged exposure to tumor blast antigens, T lymphocytes fall into a state of exhaustion and lose their ability to mount a normal cellular response. The PD-1L present on blasts in MDS promoted the transformation of cytotoxic CD4+ lymphocytes into regulatory T cells (Treg) [93]. In a study by Ozkazanca et al., the Th lymphocyte population was characterized by an increased presence (up-regulation) of PD-1, CTLA-4, TIM-3 and LAG3 receptors, attenuated secretion of IL-2, TNF-α and IFN-c, and a reduced proliferative potential [69]. In summary, MDS cells have the ability to induce inflammation which, once it enters the chronic phase, leads to an immunosuppressive microenvironment that promotes tumor growth.

## 10. The Importance of PD-1 and PD-L1 Signaling Pathway in the Course of MDSs

An association between the presence of PD-1, PD-L1 and PD-L2 and disease progression has been described in the course of many cancers [94]. It is believed that MDSs can be included in the above group, although not all research results are clear at this point. In MDSs, an increased presentation of PD-1 has been shown on effector cells and Treg cells and PD-L1 on CD34+ myeloblast. However, this was only in cases in which their percentage in the bone marrow was >5%. According to the authors of the above reports, the presence of PD-L1 correlated positively with higher IPSS-R scores [95]. However, a study by Sallman et al. on a larger population (40 vs. 107) did not confirm these reports. PD-L1 was described on more mature myeloblasts and those expressing CD38+. It was associated with a poorer prognosis. PD-L1+ blasts were characterized by a higher expression of Ki67, cyclin D1, D2 and D3 and more dynamic growth compared to PD-L1-. The presence of TP53 mutations significantly increased the presence of PD-L1 in blasts, impairing the ability to mount a local cytotoxic response by creating an immunosuppressive microenvironment. In the myeloid niche of patients with TP53 mutations, a reduced number of Tc and Th lymphocytes and an increased number of Treg lymphocytes were observed compared to the population with the wild type. Thus, it is possible that deregulation of the PD-1/PD-L1 pathway is one mechanism that worsens the prognosis of patients with TP53 mutations. Interestingly, high-risk MDSs had stronger CD274 expression than AML after transformation from MDS [95,96,97,98,99]. Stronger PD-1 expression is associated with higher patient age and risk, as determined by the IPSS score, but is not associated with MDS severity [100,101,102]. 

MDS cells are able to perversely exploit one of the most important mediators of the anti-cancer immune response: IFN-γ. Tumor blasts, despite having the ability to present costimulatory molecules that stimulate a normal response from Tc cells (CD80, CD86 and B7-H2), avoid a cytotoxic response by acquiring secondary immunity mediated by IFN-γ. The aforementioned interleukin, secreted mainly by T and NK lymphocytes, causes an increased expression of PD-L1 and PD-L2 on cells in the MDS microenvironment. Other adverse effects of IFN-γ include the activation of the proleukemogenic STAT3 pathway and the ability to accelerate the apoptosis of normal hematopoietic cells in a low-risk MDS (Lr-MDS) [77,103,104].

The standard of treatment for HR-MDS are HMAs. Unfortunately, a common phenomenon observed in clinical practice is the emergence of resistance to the use of the above group of drugs. A study of 124 patients by Yang et al. (including 69 diagnosed with MDS) showed that hypomethylation of the pdcd-1 gene occurred on leukemic myeloblasts from both patients and cell lines that were exposed to decitabine. Its expression positively correlated with drug dose and negatively correlated with OS. In the cohort of patients treated with HMA, the expression of PD-L1, PD-L2 and PD-1 was increased compared to the untreated cohort and was significantly higher in the refractory group than in those responding well to therapy. Based on the results presented above, it was concluded that the PD-1/PD-L1 pathway may be an important element in the development of HMA resistance, and that the use of its inhibitors may be effective in overcoming HMA resistance [102,105]. This became the basis for beginning clinical trials with molecules that inhibit the PD-1 receptor and its ligands.

## 11. Molecules That Inhibit the PD-1/PD-L1 Pathway and Their Use in the Treatment of MDSs

The first trials of PD-1 receptor inhibition for the treatment of patients with myeloid malignancies began as early as 2008. The Phase I study used the humanized IgG1 monoclonal antibody pidilizumab (CT-011). Among the participants in the study, only one patient was diagnosed with MDS, and in his case there was no response to treatment [101]. According to available data, there have been no repeated attempts to treat MDS with pidilizumab.

In later years, the efficacy of other PD-1 inhibitors, such as nivolumab and pembrolizumab, in breaking HMA resistance were studied. Their results differed significantly and negatively from predictions based on in vivo studies. In a Phase Ib study (NCT01953692) that used pembrolizumab as monotherapy among 28 patients enrolled in the trial with a median follow-up of 5.6 months, the ORR was only 4% and the OS at 24 months was 49% (for IPSS intermediate-1 89%, intermediate-2 22% and high-29%). Hematologic improvement was observed in only 11% of patients [106].

In the Phase II study by Garcia-Manero et al., 35 patients who lost their response to HMA were divided into two groups. One group received nivolumab (PD-1 inhibitor) as monotherapy and the other received ipilimumab (CTLA-4 T-cell antigen-4 inhibitor) (15 vs. 20). The ORRs were 13% and 35%, respectively, with an acceptable toxicity profile (the most common complications were skin rash and fatigue). In the nivolumab group, no patient achieved CR, and in the ipilimumab group, 15% of patients did. The median OS was 8 months [107]. The use of PD-1 inhibitors as monotherapy in refractory MDS appears to be a suboptimal treatment with unsatisfactory efficacy. However, it should be remembered that MDS after loss of response to HMA is associated with a poor prognosis, [108] and most of the patients included in the study were in the higher risk groups according to IPSS. Based on the above results, it can be speculated that more potential lies in inhibitors of the “similar” CTLA-4 receptor. However, this hypothesis requires further research. An attempt was made to use nivolumab in HrMDS/AML patients after alloHSCT (trial NCT04361058) as it was suspected that PD-1 blockade could support the efficacy of graft versus leukemia response. However, the trial was discontinued due to difficulties recruiting patients, and the results have not been published. The ongoing NCT03286114 trial is evaluating the efficacy of pembolizumab in treating relapsed MDS, AML or acute lymphoblastic leukemia after HSCT. 

Other researchers have focused on determining the safety and potential benefits of combining PD-1/PD-L1 inhibitors and other drugs—most commonly HMAs. In one Phase II study, AZA was used in combination with pembrolizumab in 17 previously untreated patients. The ORR was 76%, with CR reaching 18%. The median overall survival was not reached after a median follow-up of 12.8 months. For the cohort with failure after HMA (*n* = 20), the ORR was 25% and the CR rate was 5%; for the cohort with a median follow-up of 6 months, it was 5.8 months. The most common side effects were pneumonia (32%), joint pain (24%) and constipation (24%) [109]. In the study by Garcia Manero et al. discussed above, one cohort (20 patients) consisted of previously untreated patients diagnosed with MDS who were treated with a combination of nivolumab and AZA. An ORR of 75% and a CR of 50% were achieved [107]. The results of the above studies are significantly better than with PD-1/PD-L1 inhibitors in monotherapy, but it should be kept in mind that they were conducted on a previously untreated patient population.

**Figure 2:**
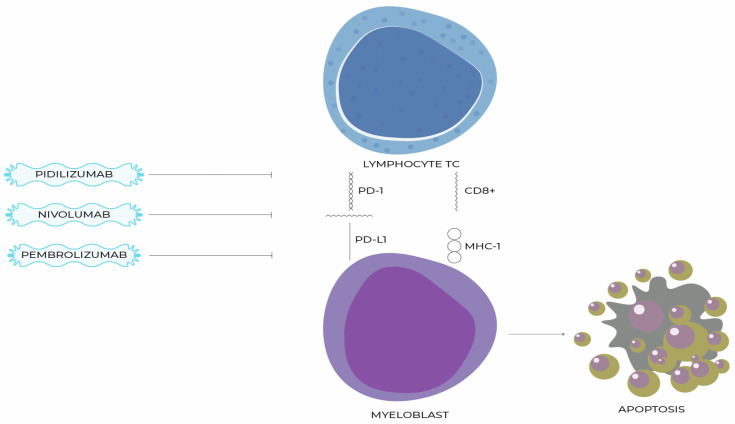
Recently tested particles with potential to inhibit PD-1/PD-L1 pathway and supporting cell apoptosis.

In an attempt to establish the potential of PD-L1 as a drug target for the treatment of MDS, a randomized, multicenter Phase II trial was conducted on 84 patients with untreated intermediate-, high- and very high-risk MDS. The study population was divided into two cohorts of 42 patients each. The former used AZA in monotherapy and the latter combined it with durvalumab. There were no statistically significant differences in ORR between treatment groups in any of the cohorts. The median OS was 11.6 vs. 16.7 months, respectively, and the PFS was 8.7 vs. 8.6 months, respectively. The treatment resulted in an increased surface expression of PD-L1 on granulocytes and monocytes but not on blasts [110]. The NCT02281084 trial, which is evaluating the efficacy of oral AZA in combination with durvalumab, is expected to conclude in December 2022. The results obtained in it may be an important addition to the existing knowledge about the effectiveness of the mentioned drug in MDS [Figure 2, Table 2].

## 12. Preclinical Data on the Association of BCL-2 Protein and the PD-1/PD-1L Pathway

In the scientific literature, there are few reports describing the interactions between the proteins of the BCL-2 family and the PD-1/PD-L1 pathway, and they are not conclusive in terms of MDSs. However, results of the available studies provided a theoretical basis for the currently conducted clinical trials. The structure of the BCL-2-associated athanogene-1 (BAG-1) protein does not allow to classify it to the BCL-2 family, even though it is closely related functionally. By binding with BCL-2, it participates in one of the pathways that inhibit apoptosis. [111] Increased expression of PD-L1 indirectly, via closed-loop signaling (epidermal growth factor receptor (EGFR)/extracellular single-regulated kinase (ERK)/PD-L1/BAG-1), induces BAG-1, which promotes phosphorylation and ubiquitination of anti-apoptotic BIM. [112] In a mouse model of chronic graft-versus-host disease (cGVHD), the knockdown of PD-L1 on neoplastic cells increased levels of BCL-2 and MCL-1 but decreased BAX and BAK in T cells, increasing their survival and the intensity of cGVHD. [113] The clinical implications of this phenomenon are difficult to predict. On one hand, the combination of BCL-2 and PD-1/PD-1L inhibitors promotes cancer cell death; on the other hand, it may result in the shortened survival of T lymphocytes. In melanoma patients, a high level of BIM in lymphocytes that show activity against tumorigenic melanocytes is a predictive marker of a good response to PD-1 inhibitors [114].

To the authors’ knowledge, no in vivo studies on the efficacy of the combination of BCL-2 inhibitors and the PD-1/PD-L1 pathway in MDS are currently available. A Phase I clinical trial (NCT03969446) is currently underway, recruiting patients with MDS, and a Phase II trial (NCT04284787) is recruiting, among others, patients with AML after MDS, in whom the combination of pembrolizumab, venetoclax and decitabine is being evaluated.

## 13. Summary

The role of PD-1/PD-L1 pathway inhibitors in MDSs is currently a subject of intense interest in the scientific community. This is evidenced by the number of clinical trials underway. However, their results are inconclusive. The results we currently have suggest the efficacy of PD-1 inhibitors in first-line treatment and a declining efficacy at relapse. Thus, it seems optimal to use targeted therapy as a first-line treatment. Perhaps the future of MDS treatment will be combination, multi-drug therapies, similar to the treatment of multiple myeloma. There are reports suggesting that venetoclax enhances the anti-tumor efficacy of PD-1 inhibitors without disrupting T-cell function [115]. The NCT03969446 trial is currently underway, evaluating the efficacy of the combination of decitabine, venetoclax and pembrolizumab in the treatment of MDS. Combining drugs that promote apoptosis and enhance the immune response with HMAs seems reasonable and promising. The trial results may help establish the clinical value of the interaction between BCL-2 and PD-1/PD-L1 family proteins.

## Figures and Tables

**Table 1:** Clinical trials for BCL-2 family proteins inhibitors in treatment of MDS.

Reference/Identifier	Trial Phase	Population Characteristics	Molecular Target	Treatment	Results/Status
Galimberti et al. [44]	Phase II Single-arm	N = 12 with MDS	BCL2L10, BAD, BAX	ATO + ascorbic acid	An increased expression of pro-apoptotic BAD and BAX and decreased anti-apoptotic BCL2L10.
McBride et al. [19]	Phase I Single-arm	N = 14 with MDS	BIM, BAK, BCL-2, MCL-1	Obatoclax	Three patients achieved improvement of transfusion dependence.
McBride et al. [19]	Phase II Single-arm	N = 24 with MDS	BIM, BAK, BCL-2, MCL-1	Obatoclax	The response rate was 8% with thrombocytopenia, anemia and pneumonia among Grades 3–4.
Wei et al. [55]	Phase IB Single-arm	N = 59 with HR-MDS	BCL-2	Venetoclax + AZA	CR = 18, mCR = 22, DS =11 and progression (PD) =2
Jilg et al. [54]	Phase IB Single-arm	N = 78 with HR-MDS	BCL-2	Venetoclax + AZA	CR = 42%, mCR = 35%
Hecker et al. [56]	Phase III Multi-arms	Ongoing	BCL-2	Venetoclax/placebo	Ongoing
NCT02966782	Phase IB Multi-arms	Ongoing	BCL-2	Venetoclax/Venetoclax + AZA	Ongoing
NCT03404193	Phase II Signle-arm	Ongoing	BCL-2	Venetoclax + Decitabine	Ongoing

**Table 2:** Clinical trials for PD-1/PD-L1 inhibitors in treatment of MDS.

Reference/Identifier	Trial Phase	Population Characteristics	Molecular Target	Treatment	Results/ Status
Garcia-Manero et al. [106]	Phase Ib Single-arm	N = 28 with MDS	PD-1	embrolizumab	ORR = 4%, OS in 24 months= 49%
Garcia-Manero et al. [107]	Phase II Multi-arms	N = 35 with MDS, lost response to HMA	PD-1/CTLA-4	Nivolumab/ipilimumab	Nivolumab: No CR, Ipilimumab CR = 15%
Chien et al. [109]	Phase II Single-arm	N = 17 with untreated MDS	PD-1	Pembrolizumab + AZA	ORR = 76%, CR = 18%.
Chien et al. [109]	Phase II Single-arm	N = 20 with MDS, lost response to HMA	PD-1	Pembrolizumab + AZA	ORR = 25%, CR = 5%.
Zeidan et al. [110]	Phase II Multi-arm	N = 84 with MDS	PD-L1	AZA/AZA + durvalumab	No statistically significant differences in ORR

## Data Availability

Data available in a publicly accessible repository.
